# Housing and Food Insecurity and Chronic Disease Among Three Racial Groups in Hawaiʻi

**DOI:** 10.5888/pcd16.180311

**Published:** 2019-01-31

**Authors:** David A. Stupplebeen

**Affiliations:** 1 University of Hawaiʻi at Mānoa, Honolulu, Hawaiʻi

## Abstract

**Introduction:**

Food and housing insecurity are social determinants of health related to chronic disease, including diabetes, cardiovascular disease (CVD), and asthma. How these insecurities affect chronic disease among the 3 largest racial groups in Hawaiʻi is unknown. The purpose of this study was to examine chronic disease by housing and food insecurity among whites, Asians, and Native Hawaiian/other Pacific Islanders (NHOPIs) in Hawaiʻi.

**Methods:**

We pooled data on 9,907 respondents from the 2009 and 2012 Behavioral Risk Factor Surveillance System. Dependent variables were diabetes, CVD, and asthma. Independent variables were housing and food insecurity. Logistic regression models were stratified by race to examine within-group differences by severity of insecurity.

**Results:**

Compared with housing secure respondents, housing insecure NHOPIs had higher adjusted odds of diabetes (odds ratio [OR] = 1.85; 95% confidence interval [CI], 1.13–3.01) and CVD (OR = 1.85; 95% CI, 1.04–3.28), and housing insecure whites (OR = 1.52; 95% CI, 1.12–2.04) and Asians (OR = 1.93; 95% CI, 1.29–2.88) had higher adjusted odds of asthma. Compared with food secure participants, food insecure NHOPIs had higher adjusted odds of diabetes (OR = 2.17; 95% CI, 1.28–3.68); food insecure whites (OR = 1.88; 95% CI, 1.16–3.05) and NHOPIs (OR = 2.04; 95% CI, 1.10–3.78) had higher adjusted odds of CVD, and food insecure whites (OR = 1.53; 95% CI, 1.06–2.22) and Asians (OR = 1.79; 95% CI, 1.05–3.06) had higher adjusted odds of asthma.

**Conclusion:**

Housing and food insecurity are associated with higher rates of chronic diseases among some races in Hawaiʻi. Policy makers should work to increase affordable housing and improve policies to increase food affordability.

**Summary**

**What is already known about this topic?**

Food and housing insecurity are related to chronic health conditions such as diabetes, cardiovascular disease, and asthma, but how these insecurities relate to chronic disease among Asians and among Native Hawaiians and other Pacific Islanders is understudied.

**What is added by this report?**

We used pooled Hawaiʻi Behavioral Risk Factor Surveillance System data among Hawaiʻi residents from 2009 and 2012 to identify differential relationships between levels of housing and food insecurity and chronic disease when stratified by racial group.

**What are the implications for public health practice?**

By understanding the relationship between food and housing insecurity by racial/ethnic group, practitioners seeking to prevent chronic disease can better address upstream determinants of health.

## Introduction

Food and housing insecurity are social determinants of health (1). Food insecurity is associated with poor physical health, diabetes, and hypertension (2,3), and housing insecurity is associated with overall poor health (4,5) and cardiovascular disease (CVD) (6). Food and housing insecurity also affect chronic disease management (7). In Hawaiʻi, over a quarter of adults were food insecure and nearly half were housing insecure in one study that used state data from 2010–2011 (8). Hawaiʻi had the highest cost of living in the nation in 2016 (9) and the second lowest median private sector wages in the country after cost of living adjustment (10). Hawaiʻi imports 85% to 90% of its food (11), and locally grown foods can be unaffordable. For example, locally grown lettuce costs from $11 to $12 a pound compared with from $4 to $6 a pound for imported lettuce (12). Oʻahu, the main population center of the state, has a chronic housing shortage (10,13).

Chronic disease is unevenly distributed among the food or housing insecure residents of Hawaiʻi. Nearly half of Hawaiʻi residents with diabetes are food insecure, and two-thirds are housing insecure. High proportions of state residents with asthma are food insecure (35.7%) or housing insecure (56.2%), and 42% of those with heart disease or a history of stroke were food insecure. More than half of Hawaiʻi residents with heart disease (53.2%) or stroke (65.9%) were housing insecure (8). Chronic disease is also unevenly distributed among the 3 largest racial groups in Hawaiʻi: Asians, Native Hawaiian/other Pacific Islanders (NHOPIs), and whites. NHOPIs have a higher prevalence of diabetes (13.1%), heart attack (4.9%), stroke (4.1%), and asthma (22.1%) than Asian or white state residents (14). A gap exists in our understanding of how food and housing insecurity are associated with chronic disease by racial group in Hawaiʻi. I hypothesized that higher food and housing insecurity were associated differently with increased odds of diabetes, CVD, and asthma across the 3 largest racial groups in Hawaiʻi.

## Methods

### Data source and sample

Cross-sectional, public use data from the 2009 and 2012 Behavioral Risk Factor Surveillance System (BRFSS) were used for this study. Methods and reliability for BRFSS items are described elsewhere (15). The Social Context Module, an optional BRFSS question set that asks about housing and income insecurity, was used during these 2 data collection periods. Data were pooled to increase the sample size of NHOPIs. Only observations from Hawaiʻi were retained, yielding an initial set of 14,265 observations. Racial groups retained for analysis were whites, Asians, and NHOPIs, leaving 13,436 observations. Chronic disease indicators were missing on 327 observations, on 2,222 observations on housing insecurity, and on 20 on food insecurity. An additional 754 observations were missing income data, and 206 were missing data on other covariates, yielding a final data set of 9,907 observations. This study used unweighted data for analysis, because the 2 survey cycles used different weights to account for cellular telephone use in 2012. This study was exempt from institutional review board review because it used secondary, publicly available data sets.

### Measures

Three chronic disease variables were used: diabetes, CVD, and asthma. All were self-reported and dichotomously recoded. Diabetes was coded from the question, “Have you ever been told by a doctor that you have diabetes?” and included only respondents who were diagnosed at age 18 or older. CVD was created by grouping any observations related to heart attack, angina, or stroke (“Has a doctor, nurse, or other health professional ever told you . . . you had a heart attack, also called a myocardial infarction/angina or coronary heart disease/stroke?”). Asthma was based on responses to the question, “Do you still have asthma?”

In addition to race, 2 more independent variables from the BRFSS Social Context Module were used for housing insecurity and food insecurity. The housing security question, “How often in the past 12 months would you say you were worried or stressed about having enough money to pay for your rent/mortgage?” was scored on a 5-point scale from “always” to “never.” The food security question, “How often in the past 12 months would you say you were worried or stressed about having enough money for nutritious meals?” used the same scale. Because of small response counts for both questions, scales were recategorized to “always/usually,” “sometimes,” and “rarely/never.”

**Covariates**. Demographic variables were sex (male/female), age, number of children in household, and marital status (married/cohabiting, divorced/separated/widowed, and single/never married). Socioeconomic variables were annual income (recoded to <$15,000, $15,000–$24,999, $25,000–$49,999, $50,000–$74,999, and ≥$75,000), education (less than high school diploma, high school graduate, some college, and college graduate or higher), and employment status (employed, unemployed, student or homemaker, retired, or unable to work). Health behaviors and indicators were binge drinking (defined as having ≥5 drinks for males or ≥4 drinks for females on one occasion in the past 30 days) (yes or no), smoking status (never smoked, quit smoking, smokes some days, smokes every day), any exercise in the past 30 days (yes or no), and last checkup by a doctor was (<12 mo, 1–<2 y, 2–<5 y, ≥5 y), and body mass index (BMI, weight in kilograms divided by height in meters squared). BMI was reclassified into 4 groups by using the calculated BMI variable from the BRFSS data set: underweight or normal weight (BMI <24.99), overweight (BMI 25–29.99), obese I (BMI 30–34.99), and obese II/III (BMI ≥35). Lastly, a dummy variable for the year data were collected was used in all models.

### Statistical analysis

Differences in level of housing and food insecurity, demographic characteristics (sex, age, children, and marriage), socioeconomic factors (income, education, and employment), and health indicators (drinking, smoking, exercise, doctor visits, and BMI) were examined by racial group by using χ^2^ tests for categorical variables and analysis of variance for continuous measures. Logistic regression models tested associations between housing and food insecurity levels with the 3 disease variables and whether associations varied significantly across racial groups and interaction between races and insecurity variables. Models were adjusted for demographic, socioeconomic, and health variables. Subsequent models stratified by race to examine the effects of housing and food insecurity differences were examined within groups. Analyses were performed by using Stata 15.1 (StataCorp). Significance was set at *P* < .05 (2-tailed).

## Results

The overall sample was 46.5% white, 39.8% Asian, and 13.7% NHOPIs (Table 1). NHOPIs had the highest prevalence of diabetes, CVD, and asthma, although Asian respondents had nearly the same prevalence of diabetes. NHOPIs also reported higher levels of housing and food insecurity than did Asian or white respondents. Differences in diabetes, asthma, and housing and food security were significant. More women participated in the survey than did men in all racial categories, and NHOPIs were younger than other respondents overall. More Asian and white respondents were married or cohabiting, whereas more NHOPIs were never married, and NHOPIs had more children than Asian or white respondents. Over a third of Asians and whites reported earning over $75,000 annually compared with slightly more than one-fifth of NHOPIs. Asian and white respondents had higher levels of educational attainment. A higher proportion of NHOPIs were unemployed or unable to work than whites or Asians, and a higher proportion of NHOPIs binged on alcohol and smoked some days or every day. NHOPIs had a higher BMI than Asians or whites. Whites were more likely to have exercised and more likely not to have visited a doctor in a year or more.

**Table 1:** Characteristics of Respondents, Study on Housing and Food Insecurity and Chronic Disease Among Three Hawaiʻi Racial Groups (N = 9,907), Behavioral Risk Factor Surveillance System, 2009 and 2012^a^

Characteristic	White	Asian	NHOPI	Total
**Total**	4,611 (46.5)	3,942 (39.8)	1,354 (13.7)	9,907 (100)
**Diabetes**	250 (5.4)	441 (11.2)	153 (11.3)	844 (8.5)
**Asthma**	413 (9.0)	297 (7.5)	223 (16.5)	933 (9.4)
**Cardiovascular disease**	354 (7.7)	272 (6.9)	116 (8.6)	742 (7.5)
**Housing insecurity**				
Always/usually	526 (11.4)	306 (7.8)	261 (19.9)	1,093 (11.0)
Sometimes	698 (15.1)	789 (20.0)	345 (25.5)	1,832 (18.5)
Rarely/never	3,387 (73.5)	2,847 (72.2)	748 (55.2)	6,982 (70.5)
**Food insecurity**				
Always/usually	263 (5.7)	137 (3.5)	169 (12.5)	569 (5.7)
Sometimes	500 (10.8)	499 (12.7)	286 (21.1)	1,285 (13.0)
Rarely/never	3,848 (83.5)	3,306 (83.9)	899 (66.4)	8,053 (81.3)
**Sex**				
Male	2,063 (44.7)	1,768 (44.9)	568 (42.0)	4,399 (44.4)
Female	2,548 (55.3)	2,174 (55.2)	786 (58.1)	5,508 (55.6)
**Age, y, mean (standard deviation)**	55.6 (15.3)	55.7 (16.7)	47.3 (16.2)	54.5 (16.2)
**Annual income, $**				
<15,000	344 (7.5)	245 (6.2)	167 (12.3)	756 (7.6)
15,000–24,999	558 (12.1)	466 (11.8)	267 (19.7)	1291 (13.0)
25,000–49,999	1,183 (25.7)	1,159 (29.4)	399 (29.5)	2,741 (27.7)
50,000–74,999	808 (17.5)	763 (19.4)	227 (16.8)	1,798 (18.2)
≥75,000	1,718 (37.3)	1,309 (33.2)	294 (21.7)	3,321 (33.5)
**Marital status**				
Married/cohabiting	2,706 (58.7)	2,414 (61.2)	744 (55.0)	5,864 (59.2)
Divorced/separated/widowed	1,281 (27.8)	854 (21.7)	289 (21.3)	2,424 (24.5)
Never married	624 (13.5)	674 (17.1)	321 (23.7)	1,619 (16.3)
**Education**				
Less than high school diploma	126 (2.7)	144 (3.7)	79 (5.8)	349 (3.5)
High school graduate	896 (19.4)	968 (24.6)	614 (45.4)	2,478 (25.0)
Some college	1,281 (27.8)	1,111 (28.2)	367 (27.1)	2,759 (27.9)
≥College graduate	2,308 (50.1)	1,719 (43.6)	294 (21.7)	4,321 (43.6)
**Children in household**	0.5 (.9)	0.6 (1.0)	1.1 (1.4)	0.6 (1.0)
**Employment status**				
Employed	2,733 (59.3)	2,335 (59.2)	851 (62.9)	5,919 (59.8)
Unemployed	209 (4.5)	150 (3.8)	97 (7.2)	456 (4.6)
Student/homemaker	282 (6.1)	188 (4.8)	101 (7.5)	571 (5.8)
Retired	1,204 (26.1)	1,207 (30.6)	226 (16.7)	2637 (26.6)
Unable to work	183 (4.0)	62 (1.6)	79 (5.8)	324 (3.3)
**Binge drinker (yes)^b^**	746 (16.2)	506 (12.8)	317 (23.4)	1,569 (15.8)
**Smoking status**				
Never smoked	2,331 (50.6)	2,475 (62.8)	685 (50.6)	5,491 (55.4)
Quit	1646 (35.7)	1,035 (26.3)	395 (29.2)	3,076 (31.1)
Smokes some days	222 (4.8)	118 (3.0)	80 (5.9)	420 (4.2)
Smokes every day	412 (8.9)	314 (8.0)	194 (14.3)	920 (9.3)
**Any exercise in 30 days (yes)**	3,976 (86.2)	3,101 (78.7)	1,083 (80.0)	8,160 (82.4)
**Body mass index^c^**				
Underweight/normal weight	2,058 (44.6)	1,927 (48.9)	278 (20.5)	4,263 (43.0)
Overweight	1,594 (34.6)	1,384 (35.1)	450 (33.2)	3,428 (34.6)
Obese I	605 (13.1)	443 (11.2)	313 (23.1)	1,361 (13.7)
Obese II/III	354 (7.7)	188 (4.8)	313 (23.1)	855 (8.6)
**Last doctor visit**				
<12 mo	2,813 (61.0)	2,750 (69.8)	859 (63.4)	6,422 (64.8)
1 y–<2 y	762 (16.5)	526 (13.3)	221 (16.3)	1,509 (15.2)
2 y–<5 y	515 (11.2)	336 (8.5)	141 (10.4)	992 (10.0)
≥5 y	521 (11.3)	330 (8.4)	133 (9.8)	984 (9.9)

Abbreviation: NHOPI, native Hawaiian/other Pacific Islander.

a Categorical variables tested via χ^2^ test; continuous variables tested via analysis of variance. Values are number (percentage) unless otherwise indicated. All variables were significant at *P* < .001 except cardiovascular disease (*P* = .11) and sex (*P* = .15).

b Binge drinking defined as having ≥5 drinks for males or ≥4 drinks for females on one occasion in the past 30 days.

c Body mass index (weight in kg/height in m^2^) categories: underweight/normal weight = <24.99; overweight = 25–29.99; obese I = 30–34.99; obese II/III ≥35.

Significant differences between races in all disease conditions by housing and food insecurity were found (Table 2). When examining differences in disease within a racial group by level of insecurity, only a few conditions were still significantly different. Regarding housing insecurity, diabetes was only significant for NHOPIs, asthma was significant for all groups, and CVD was significant only for Asian respondents. Regarding food security, diabetes was significant only for white respondents and NHOPIs, and CVD was significant only for whites. Asthma was significant for all groups.

**Table 2:** Prevalance of Chronic Disease Among Respondents (N = 9,907), Study of Housing and Food Insecurity and Chronic Disease Among Three Hawaiʻi Racial Groups (N = 9,907), Behavioral Risk Factor Surveillance System, 2009 and 2012^a^

Chronic Disease	White	Asian	NHOPI	Total
**Overall**	4,611 (46.5)	3,942 (39.8)	1,354 (13.7)	9,907 (100)
**Housing Insecurity**				
**Diabetes**	250 (5.4)	441 (11.2)	153 (11.3)	844 (8.5)
Always/usually	33 (13.2)	27 (6.1)	40 (26.1)	100 (11.9)
Sometimes	28 (11.2)	80 (18.1)	26 (17.0)	134 (15.9)
Rarely/never	189 (75.6)	334 (75.7)	87 (56.9)	610 (72.3)
*P* value^b^	.16	.18	.01	<.001
**CVD**	354 (7.7)	272 (6.9)	116 (8.6)	742 (7.5)
Always/usually	40 (11.3)	17 (6.3)	29 (25.0)	86 (11.6)
Sometimes	47 (13.3)	39 (14.3)	28 (24.1)	114 (15.4)
Rarely/never	267 (75.4)	216 (79.4)	59 (50.9)	542 (73.1)
*P* value^b^	.58	.02	.26	<.001
**Asthma**	413 (9.0)	297 (7.5)	223 (16.5)	933 (9.4)
Always/usually	84 (20.3)	41 (13.8)	55 (24.7)	180 (19.3)
Sometimes	73 (17.7)	78 (26.3)	63 (28.3)	214 (22.9)
Rarely/never	256 (62.0)	178 (59.9)	105 (47.1)	539 (57.8)
*P* value^b^	<.001	<.001	.02	<.001
**Food Insecurity**				
**Diabetes**	250 (5.4)	441 (11.2)	153 (11.3)	844 (8.5)
Always/usually	24 (9.6)	16 (3.6)	31 (20.3)	71 (8.4)
Sometimes	27 (10.8)	49 (11.1)	23 (15.0)	99 (11.7)
Rarely/never	199 (79.6)	376 (85.3)	99 (64.7)	674 (79.9)
*P* value^b^	.02	.58	<.01	<.001
**CVD**	354 (7.7)	272 (6.9)	116 (8.6)	742 (7.5)
Always/usually	33 (9.3)	8 (2.9)	22 (19.0)	63 (8.5)
Sometimes	38 (10.7)	23 (8.5)	25 (21.6)	86 (11.6)
Rarely/never	283 (79.9)	241 (88.6)	69 (59.5)	593 (79.9)
*P* value^b^	<.01	.08	.07	<.001
**Asthma**	413 (9.0)	297 (7.5)	223 (16.5)	933 (9.4)
Always/usually	52 (12.6)	21 (7.1)	38 (17.0)	111 (11.9)
Sometimes	63 (15.3)	41 (13.8)	51 (22.9)	155 (16.6)
Rarely/never	298 (72.2)	235 (79.1)	134 (60.1)	667 (71.5)
*P* value^b^	<.001	<.01	.04	<.001

Abbreviation: NHOPI, native Hawaiian/other Pacific Islander.

a Values are number (percentage) unless otherwise indicated.

b Assessed by χ^2^ test.

In initial logistic regression models, the association between race and levels of housing or food insecurity varied by diabetes, CVD, and asthma (Table 3). The fully adjusted model controlled for socioeconomic factors, demographics, and health indicators. Interaction effects between race and each of the insecurity variables was significant for all fully adjusted models (*P* < .01). In the racially stratified models, as insecurity increased, odds of each chronic disease increased, although some notable differences and nonsignificant odds were found.

**Table 3:** Crude and Adjusted Logistic Regression Models, Study of Housing and Food Insecurity and Chronic Disease Among Three Hawaiʻi Racial Groups (N = 9,907), Behavioral Risk Factor Surveillance System, 2009 and 2012^a^

Chronic Disease	White	Asian	NHOPI
**Housing Insecurity Models**			
**Diabetes unadjusted**			
Never/rarely	1 [Reference]		
Sometimes	0.72 (0.48–1.07)	0.85 (0.66–1.10)	0.62 (0.39–0.98)
Always/usually	1.16 (0.79–1.08)	0.74 (0.49–1.12)	1.45 (0.96–2.18)
**Diabetes adjusted**			
Never/rarely	1 [Reference]		
Sometimes	0.85 (0.55–1.34)	1.29 (0.97–1.73)	0.76 (0.46–1.26)
Always/usually	1.19 (0.75–1.88)	1.01 (0.63–1.61)	1.85 (1.13–3.01)
**Cardiovascular disease unadjusted**			
Never/rarely	1 [Reference]		
Sometimes	0.84 (0.61–1.16)	0.63 (0.45–0.90)	1.03 (0.65–1.65)
Always/usually	0.96 (0.68–1.36)	0.71 (0.43–1.18)	1.46 (0.91–2.34)
**Cardiovascular disease adjusted**			
Never/rarely	1 [Reference]		
Sometimes	1.27 (0.89–1.82)	1.05 (0.71–1.53)	1.61 (0.95–2.73)
Always/usually	1.19 (0.79–1.81)	1.24 (0.70–2.19)	1.85 (1.04–3.28)
**Asthma unadjusted**			
Never/rarely	1 [Reference]		
Sometimes	1.43 (1.09–1.88)	1.65 (1.25–2.18)	1.35 (0.97–1.92)
Always/usually	2.32 (1.78–3.03)	2.35 (1.63–3.38)	1.61 (1.12–1.92)
**Asthma adjusted**			
Never/rarely	1 [Reference]		
Sometimes	1.21 (.91–1.62)	1.51 (1.11–2.04)	1.43 (.99–2.07)
Always/usually	1.52 (1.12–2.04)	1.93 (1.29–2.88)	1.25 (.83–1.90)
**Food Insecurity Models**			
**Diabetes unadjusted**			
Never/rarely	1 [Reference]		
Sometimes	1.06 (0.70–1.61)	.86 (0.63–1.17)	0.71 (0.44–1.14)
Always/usually	1.88 (1.21–2.94)	1.06 (0.62–1.81)	1.86 (1.19–2.90)
**Diabetes adjusted**			
Never/rarely	1 [Reference]		
Sometimes	.88 (.55–1.43)	1.23 (0.86–1.76)	0.86 0(.51–1.50)
Always/usually	1.44 (.83–2.49)	1.34 (0.73–2.46)	2.17 (1.28–3.68)
**Cardiovascular disease unadjusted**			
Never/rarely	1 [Reference]		
Sometimes	1.04 (.73–1.48)	0.61 (0.40–0.95)	1.15 (0.71–1.86)
Always/usually	1.81 (1.23–2.65)	0.78 (0.38–1.62)	1.80 (1.08–3.00)
**Cardiovascular disease adjusted**			
Never/rarely	1 [Reference]		
Sometimes	1.06 (.70–1.60)	0.91 (0.56–1.48)	1.66 (0.96–2.88)
Always/usually	1.88 (1.16–3.05)	1.15 (0.56–1.48)	2.04 (1.10–3.78)
**Asthma unadjusted**			
Never/rarely	1 [Reference]		
Sometimes	1.72 (1.29–2.29)	1.18 (0.83–1.66)	1.24 (0.87–1.76)
Always/usually	2.94 (2.11–4.07)	2.40 (1.48–3.90)	1.65 (1.10–2.47)
**Asthma adjusted**			
Never/rarely	1 [Reference]		
Sometimes	1.12 (.81–1.54)	1.03 (0.71–1.50)	1.08 (0.73–1.59)
Always/usually	1.53 (1.06–2.22)	1.79 (1.05–3.06)	1.16 (0.73–1.83)

Abbreviation: NHOPI, native Hawaiian/other Pacific Islander.

a Unadjusted models of insecurity and data collection period only. Adjusted models control for demographic and socioeconomic characteristics (sex, income, education, marital status, number of children in household), behavioral variables (binge drinking, smoking status, body mass index, any exercise in last 30 days, last doctor checkup), and year. Values are odds ratio (95% confidence interval).

**Diabetes.** In the unadjusted model, respondents reporting any housing insecurity did not have higher odds of diabetes than those who were housing secure. After controlling for other demographic, socioeconomic, and health indicators, NHOPIs with high housing insecurity had significantly higher odds (OR = 1.85; 95% CI, 1.13–3.01) of diabetes compared with those with secure housing. In unadjusted models examining food insecurity, whites (OR = 1.88; 95% CI, 1.21–2.94) and NHOPIs (OR = 1.86; 95% CI, 1.19–2.90) who had high food insecurity had significantly higher odds of diabetes than those who were food secure; however, in fully adjusted models, the effect for whites became nonsignificant while the effect for NHOPIs increased (OR = 2.17; 95% CI, 1.28–3.68).

**Cardiovascular disease.** Among NHOPIs CVD was only associated with experiencing housing insecurity in the fully adjusted model (OR = 1.85; 95% CI, 1.04–3.28), similar to the adjusted model for diabetes. Whites (OR = 1.81; 95% CI, 1.23–2.65) and NHOPIs (OR = 1.80, 95% CI, 1.08–3.00) with higher food insecurity had higher odds of CVD than their food secure counterparts in unadjusted models. After controlling for demographics, socioeconomic factors, and health behaviors, this effect was amplified, with slightly increased odds for whites (OR = 1.88; 95% CI, 1.16–3.05) and a larger increase for NHOPIs (OR = 2.04; 95% CI, 1.10–3.78).

**Asthma**. In unadjusted models for housing insecurity, all groups except NHOPIs who were sometimes housing insecure had higher odds of asthma than their housing secure counterparts. However, in fully adjusted models for asthma, the magnitude of these effects was diminished. Asians who experienced housing insecurity sometimes (OR = 1.51; 95% CI, 1.11–2.04) or usually/always (OR = 1.93; 95% CI, 1.29–2.88) had higher odds of asthma than their housing secure counterparts. Whites who experienced high levels of housing insecurity also had significantly higher odds (OR = 1.52; 95% CI, 1.12–2.04). A similar pattern emerged in examining food insecurity. In adjusted food security models, only whites (OR = 1.53; 95% CI, 1.06–2.22) and Asians (OR = 1.79; 95% CI, 1.05–3.06) who experienced the highest level of food insecurity had higher odds of asthma than those who were food secure.

## Discussion

I hypothesized that chronic disease odds differed among the 3 largest racial groups in Hawaiʻi by level of food and housing insecurity, and I found support for this hypothesis. Only the highest levels of housing and food insecurity were associated with diabetes among NHOPIs. CVD was only associated with the highest level of housing insecurity among NHOPIs and with the highest levels of food insecurity for whites and NHOPIs. Lastly, asthma was associated with housing insecurity among Asians, with the highest level of housing insecurity among whites, and with the highest levels of food insecurity among both Asians and whites. I found no association between diabetes and housing or food insecurity for whites and Asians. Housing and food insecurity were not associated with increased odds of CVD among Asians. No relationship was found between housing and food insecurity among NHOPIs, which could be because NHOPIs have the highest asthma prevalence in the state. The odds presented here should be interpreted with caution because the number of respondents with high housing and food insecurity who also had one of the 3 chronic conditions was low, and data collection precluded weighting.

The findings in this study align with those of other studies. Mau and colleagues in their 2010 process evaluation of a pilot translation of a diabetes prevention program found food access and cost were major social influences in NHOPI food consumption (16), which could affect diabetes prevention (7) and management (17,18). Another national study exploring health indicators for NHOPIs analyzed sex and ethnic differences in meeting fruit and vegetable consumption recommendations (19) but did not assess food security to help contextualize food purchasing decisions. A different study examining diet and diabetes, but not food security, in a Hawaiʻi-based cohort found consumption of fats and meats increased likelihood for diabetes among whites and Japanese Americans, but not among Native Hawaiians, for whom the authors conclude that race and obesity may be a stronger predictor for diabetes (20). In my study, after controlling for obesity, odds of diabetes for NHOPIs who experienced food insecurity were more than twice higher than for those who were food secure, although this was not true for Asians or whites. Food insecurity is associated with CVD in other studies (21), although results in both were not stratified by race as in the present study, which found that food insecure whites and NHOPIs had higher odds of CVD. Although a positive relationship between food insecurity and asthma was found among children (3,22), little is known about this relationship among adults, although my study helps to fill this knowledge gap.

The relationship between housing insecurity and chronic disease is a less studied area, and more must be done to understand this relationship. One study conducted in Pennsylvania found no associations between housing insecurity and heart disease, diabetes, or asthma (5). Conversely, our study found associations between housing insecurity and diabetes and CVD for NHOPIs, and between housing insecurity and asthma for Asians and whites.

My study had several limitations. First, half of observations were missing on the housing insecurity question because it was asked only of those who own or rent their home. A χ^2^ test examining differences between those missing and included in the analyses found no differences in chronic disease and housing insecurity by racial group. Highly significant differences were found between nonmissing and missing whites and Asians on food security, meaning the resulting logistic regression models’ odds for these 2 racial groups on food insecurity may be underestimated. Another limitation is that this data set was not longitudinal and cannot explain whether housing or food insecurity contributed to disease or vice versa. This study used unweighted data, because weights changed between 2009 and 2012 to account for cellular telephone use. As such, the results cannot be generalized to the state’s population, nor are they generalizable to residents of the continental United States.

The Social Context Module also had limitations. In 2012, the second time Hawaiʻi used the module, hypertension data were not collected. Aligning collection of module and hypertension questions would allow exploration of the relationship between these factors. The module should be used more frequently to better understand how housing and food insecurity may currently affect Hawaiʻi residents given the state’s high cost of living (9). Another recommendation would be to use questions drawn from other surveys, such as the Household Food Insecurity Scale (18) or the US Census American Housing Survey, which ask direct questions about missed rent or mortgage payments and evictions (23).

This study used the racial categorizations provided by BRFSS, which are based on Office of Management and Budget classifications (24). Ethnicity plays a bigger role in economic and social stratification in Hawaiʻi than in continental United States racial constructs. Traditionally, the Hawaiʻi state health department uses ethnic variables (eg, Chinese or Japanese rather than a pan-Asian variable as is used in BRFSS) to account for ethnic variability, historical context, and emergent immigration patterns (24). Using generic groups like Asian and NHOPI as I did in this study masks ethnic and cultural variability and group differences (25). More must be done to expand the literature on how different ethnic groups within heterogeneous categories like Asian or NHOPI experience housing and food insecurity.

Numerous research questions remain unanswered by this study, including the possible influence of gender differences on housing and food insecurity by racial/ethnic groups. More must be done to understand the policy mechanisms behind food and housing insecurity, because these 2 factors may augment or reinforce each other (26). In the Hawaiʻi context, yet to be studied are the relationship between chronic disease and the state’s overdependence on imported food, the state’s commercial crops, the pressure to develop state lands because of their high value (11,13), and the impact of the Jones Act (also known as the Maritime Trade Act of 1920) on food costs (11). By increasing our understanding of how these contexts relate to health, we may better inform policy and social contexts that can improve decision making about health, as described in Frieden’s Health Impact Pyramid (27). Policy makers should foster partnerships with business to encourage consideration of health outcomes in business decision making. The state should prioritize food affordability as a way to assist diabetes prevention (7) and management (17,18).

Hawaiʻi should also increase affordable housing development. One report by a nonprofit group estimated that the state needs 64,000 new affordable housing units by 2020 to keep pace with demand and recommended rental subsidies to help ameliorate housing insecurity faced by renters (10). For Native Hawaiians in particular, health is related to the state’s history of colonization, institutionalized racism, and discrimination (28). One possible way to alleviate housing insecurity for Native Hawaiians, which this study found was related to diabetes and CVD, would be to increase distribution of Hawaiian Home Lands parcels (29,000 people are currently on the homestead waitlist) (29), or to encourage development of high-density housing on an urban homeland parcel for Native Hawaiians (30).

My study described the relationship between housing and food insecurity and chronic disease in Hawaiʻi, stratified by the 3 largest racial groups in the state, whites, Asians, and NHOPIs. These analyses showed that among NHOPIs, food and housing insecurity were related to diabetes and CVD prevalence. Housing and food insecure Asians and whites had higher odds of asthma, and food insecure whites had higher odds of CVD than their food secure counterparts. More research is needed to understand how race, food, and housing insecurity interact and contribute to chronic disease. Governments and state agencies should enact policies that ameliorate food and housing insecurity to create a healthy environment.
